# Enhancing soil amendment for salt stress using pretreated rice straw and cellulolytic fungi

**DOI:** 10.1038/s41598-024-64705-1

**Published:** 2024-06-17

**Authors:** Yen Nhi Ma, Wiyada Mongkolthanaruk, Nuntavun Riddech

**Affiliations:** Department of Microbiology, Faculty of Science, Khon Kaen University, Khon Kaen, 40002 Thailand

**Keywords:** Cellulolytic fungi, Pretreated rice straw, Soil amendment, Salt stress, Microbiology, Applied microbiology, Environmental microbiology

## Abstract

Rice straw breakdown is sluggish, which makes agricultural waste management difficult, however pretreatment procedures and cellulolytic fungi can address this issue. Through ITS sequencing, *Chaetomium globosum* C1, *Aspergillus* sp. F2, and *Ascomycota* sp. SM2 were identified from diverse sources. *Ascomycota* sp. SM2 exhibited the highest carboxymethyl cellulase (CMCase) activity (0.86 IU/mL) and filter-paper cellulase (FPase) activity (1.054 FPU/mL), while *Aspergillus* sp. F2 showed the highest CMCase activity (0.185 IU/mL) after various pretreatments of rice straw. These fungi thrived across a wide pH range, with *Ascomycota* sp. SM2 from pH 4 to 9, *Aspergillus* sp. F2, and *Chaetomium globosum* C1 thriving in alkaline conditions (pH 9). FTIR spectroscopy revealed significant structural changes in rice straw after enzymatic hydrolysis and solid-state fermentation, indicating lignin, cellulose, and hemicellulose degradation. Soil amendments with pretreated rice straw, cow manure, biochar, and these fungi increased root growth and soil nutrient availability, even under severe salt stress (up to 9.3 dS/m). The study emphasizes the need for a better understanding of *Ascomycota* sp. degradation capabilities and proposes that using cellulolytic fungus and pretreatment rice straw into soil amendments could mitigate salt-related difficulties and improve nutrient availability in salty soils.

## Introduction

The requirement for agricultural sustainability in the face of severe global concerns emphasizes the importance of novel soil development technologies. Among these difficulties, soil salinity stands out as a sincere concern, impacting around 20% of crops globally^1^. High salt levels in soils reduce root water uptake and nutrient absorption, resulting in lower agricultural production and stunted plant development. Furthermore, salinity stress can change crop composition, causing the buildup of toxic ions such as sodium, making crops unfit for ingestion. Addressing the negative consequences of salinity necessitates a diverse strategy, which includes the creation of salt-tolerant agricultural types and soil remediation techniques.

Soil amendments such as rice straw and biochar, serve a key role in enriching soil with organic carbon and nitrogen, which is essential for modern agricultural techniques. This entails purposely modifying soil qualities to enhance their physical, chemical, and biological features, which are vital for countering soil salinity^2^. In saline soils, utilizing rice straw as a soil amendment is a comprehensive method to alleviate salinity’s harmful impacts in paddy fields^3^. Additionally, soil additives can improve the soil’s cation exchange capacity, allowing for improved nutrient retention and release. These materials disintegrate slowly, allowing for the steady release of critical nutrients over time while also improving soil structure, nitrogen cycling, and pH stability. Furthermore, biochar helps minimize nutrient leaking into groundwater and surface water bodies, addressing a frequent problem linked with salt stress and traditional fertilization approaches^4^. The use of soil amendment could reduce reliance on inorganic fertilizer and mitigate chemical residues in the soil may result from intensive agriculture’s tendency to prioritize short-term profits over long-term soil health in fertilization management^5^. The application of soil amendments such as rice straw and biochar to soils with high organic content enhances nutrient absorption and enzyme activity, thereby mitigating the problems associated with the overuse of fertilizers^2^.

Rice straw is one of the most prevalent agricultural wastes in the world, yielding 1–1.5 kg per kilogram of produced grain^6^. Rice straw is rich in critical elements such as nitrogen (40%), phosphorus (30%), potassium (80%), sulfur, and calcium, as well as cellulose (32–37%), hemicellulose (29–37%), and lignin (5–15%)^3,7^. However, the usual practice of removing rice straw from fields, which commonly ends in its burning, not only causes nutritional loss but also contributes to environmental pollution, increasing air quality and respiratory concerns.

In this regard, using microorganisms to decompose rice straw appears to be a promising method. Cellulolytic fungi, which have unique enzyme activity capable of degrading complex organic molecules such as cellulose, play a significant role in soil ecosystems. Enzymes including endoglucanase, cellobiohydrolase, and β-glucosidases help break down cellulose, facilitating fungal development^8^. Noteworthy fungus such as *Aspergillus*, *Chaetomium*, and *Trichoderma* have been shown to improve straw decomposition in the field^9^. Furthermore, fungi found in the plant rhizosphere, such as *Aspergillus*, *Penicillium*, and *Trichoderma*, promote plant growth by producing enzymes, inorganic acids and phosphatases, which aid in the solubilization and mineralization of organic phosphorus^10,11^.

However, the slow degradation rate of lignin and hemicellulose structures in rice straw is a considerable problem for composting procedures. To remedy this, pretreatment treatments, whether chemical or biological, can degrade inhibitory compounds, making rice straw more accessible to cellulolytic fungus. Chemical pretreatment using strong alkali or inorganic acids is often regarded as one of the most efficient pretreatment procedures^12^. This increases nutrient availability and boosts fungal proliferation and activity, shortening decomposition time, which is critical for agricultural and industrial applications that need quick turnover. Previous investigations have shown that pretreatment with NaOH efficiently enhances enzyme accessibility to cellulose while eliminating lignin and a minor proportion of hemicelluloses^13^. Peng et al. and Obi et al.^12,14^ reported on the compositional and structural alterations of rice straw using diluted acetic acid as a green pretreatment. Understanding the symbiotic interaction between cellulolytic fungi and soil amendments is critical to maximizing their potential in sustainable agriculture. Organic agricultural and industrial wastes can be efficiently bioconverted using solid-state fermentation (SSF), a microbial fermentation technology that is used to insoluble materials with little free-flowing liquid^15^). SSF is considered as a necessary and feasible food processing technique for transforming organic agricultural and industrial waste^16^. Indeed, SSF is particularly successful in converting diverse agro-lignocellulosic substrates—such as straws, husks, and bagasse—into useful industrial products^14^. Importantly, the microorganisms involved in SSF, particularly filamentous fungus, have been declared harmless, resulting in finished products devoid of toxins and acceptable for consumption by animals and humans^15^. As Oiza et al.^17^ point out, this strategy provides a long-term option for recycling agricultural waste, including rice straw. Their findings highlighted SSF’s potential to promote biotechnology and boost global competitiveness due to its efficiency and cost-effectiveness. Understanding and utilizing SSF processes encourages innovation and increases market competitiveness in the organic fertilizer business through biotechnology applications, while also supporting sustainable agriculture practices and reduce agro-wastes. Therefore, this study focused on enhancing soil amendment and carbon sequestration efforts by incorporating pre-treated rice straw and biochar as primary inputs. We demonstrated that cellulolytic fungi can effectively break down rice straw and its substrates through SSF. Our research also highlighted the use of these substrates as soil amendments to promote rice seedling growth under saline environments, thus promoting sustainable agricultural practices.

## Results

### Isolation fungi and bacteria from different samples

Four distinct kinds of samples were collected and measured for pH and EC values as shown in Table 1. Sixteen isolates were isolated from 4 kinds of samples: 6 from heavy saline soil (SH), 5 from semi-saline soil (SM), 2 from carbon powder (C) source, and 3 from filter cake powder (F). Furthermore, these isolates were selected to investigate their cellulase activity in a subsequent study.

**Table 1:** The pH and EC value of 4 distinct kinds of soil sample.

Sample	pH	EC (dS/m)
Heavy saline soil (SH)	6.99 ± 0.18	4.05 ± 0.47
Semi saline soil (SM)	6.05 ± 0.26	2.03 ± 0.02
Carbon powder (C)	8.93 ± 0.13	2.56 ± 0.07
Filter cake powder (F)	6.48 ± 0.05	5.13 ± 1.61

### Qualitative screening of cellulase-producing isolate and plant growth promoting activities

All 16 fungal isolates underwent screening for endoglucanase activity on CMC agar plates using the Congo red technique. Approximately 8 isolates exhibited zones of hydrolysis on the CMC agar plates, indicating a positive result for cellulase activity (Table 2). Additionally, these isolates were assessed for various plant growth-promoting activities, including phosphate solubilization, potassium solubilization, and indole-3-acetic acid (IAA) production. Isolate SM2, SM4, and SH1 demonstrated the capability to dissolve phosphate, while only isolate SM2 exhibited the ability to solubilize potassium. Regarding IAA production, all isolates exhibited a range of IAA production levels, spanning from 0.03 to 8.36 µg/mL. This indicates that the majority of isolates possessed the capacity to produce IAA, a valuable plant growth-promoting hormone.

**Table 2:** Relative enzyme activity index (I_CMC_) and plant growth promoted by outstanding isolates.

Isolates	Relative enzyme activity index (I_CMC_)	Phosphate solubilization (SI)	Potassium solubilization (SI)	IAA production (μg/mL)
C1	1.2 ± 0.06	–	–	8.36 ± 0.50
F2	1.02 ± 0.00	–	–	6.24 ± 0.81
SM1	1.12 ± 0.01	–	–	4.21 ± 0.40
SM2	1.80 ± 0.05	2.07 ± 0.01	2.22 ± 0.05	3.60 ± 0.50
SM4	1.36 ± 0.20	2.15 ± 0.05	–	2.84 ± 0.31
SM5	1.18 ± 0.01	–	–	3.24 ± 0.51

Noted that (–) negative result.

### The effect of varying pH on isolate growth

**Figure 1:**
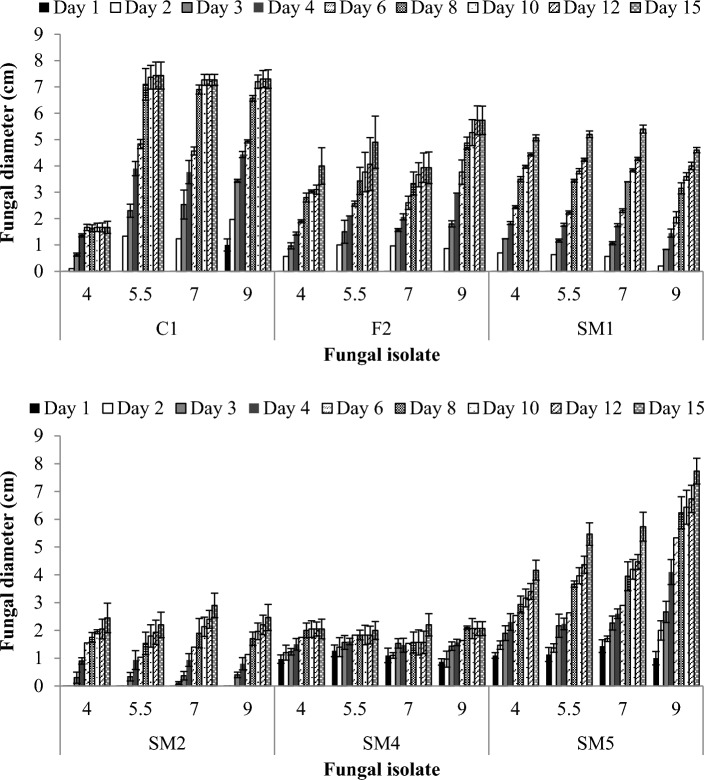
Growth (colony expansion) of 6 selected fungal isolates on various pH values on NA medium. Measurements are the mean of three diameter measurements for at least three replication and error bars indicate the standard error of the mean.

The presented data sheds light on fungal biomass and diameter growth across different pH conditions (pH 4 to 9) for a range of fungal isolates (SM1, SM2, SM4, SM5, C1, and F2). Understanding how pH influences fungal biomass growth is pivotal for assessing fungal adaptability and performance in diverse environments. In Fig. 1, the results revealed the influence of different pH values (4, 5.5, 7, and 9) on the diameter growth of six fungal isolates over 15 days. Isolates SM1, SM2, and SM4 exhibited consistent growth diameters across all tested pH values. These isolates demonstrated no significant differences in their responses to varying pH conditions (P ≤ 0.05). In contrast, isolates C1, F2, and SM5 displayed favorable growth under alkaline conditions (pH 9) when compared to neutral pH (pH 5.5). However, isolated C1 faced inhibition at pH 4. It’s evident that fungal biomass increases as the pH transitions from acidic (pH 4) towards neutral (pH 7), with potential variations occurring under alkaline conditions (pH 9) (Fig. 1). Isolates SM1, SM2, and F2 achieved the highest biomass production after 14 days at pH 7 compared to pH 4 and pH 9. Under alkaline conditions (pH 9), isolate C1 and SM5 displayed superior biomass production, while isolate SM4 thrived in acidic conditions. The result suggested that these isolates were in various experimental settings, highlighting their versatility and adaptability to diverse pH environments.

### Endoglucanase (CMCase) activity and filter paper (FPase) activity of fungal isolate

**Figure 2:**
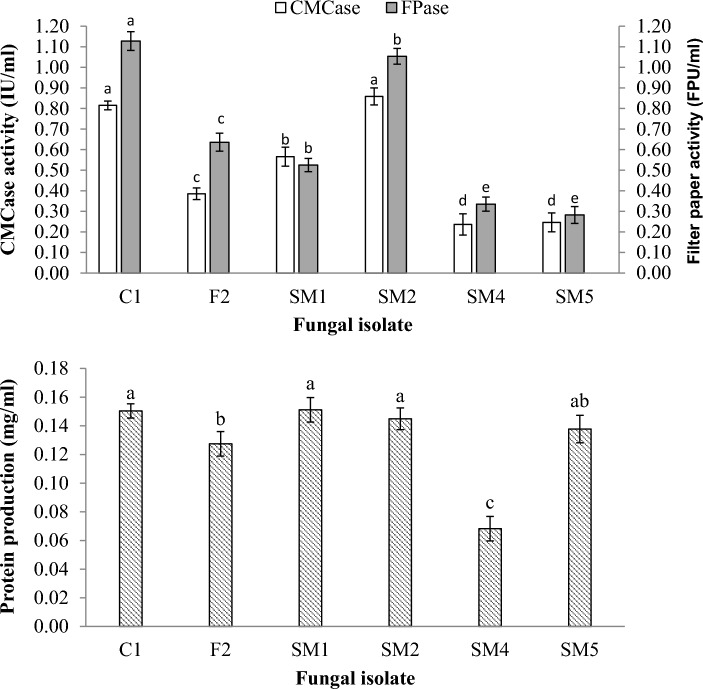
The enzyme activities and protein concentration produced by fungal isolates. The express as mean value ± SDTEV, the small letters indicated significant differences in values of each fungal isolate (by LSD, P ≤ 0.05).

In this study, we investigated the enzyme activity of six fungal isolates—C1, F2, SM1, SM2, SM4, and SM5—specifically focusing on two key enzymes: CMCase (Carboxymethyl Cellulase) and Fpase (Filter Paper cellulase). These enzymes play pivotal roles in cellulose degradation, an essential process for breaking down complex plant materials. Among them, isolate SM2 displayed the highest CMCase activity, measuring at 0.86, followed by isolates C1 (0.82 IU/mL) and SM1 (0.57 IU/mL) (Fig. 2). In contrast, isolates SM4 (0.24 IU/mL), SM5 (0.25 IU/mL), and F2 (0.39 IU/mL) exhibited lower CMCase activity. FPase activity also exhibited variability across the fungal isolates. Isolate SM2 demonstrated the highest FPase activity at 1.054 FPU/mL, followed by isolates C1 (1.128 FPU/mL) and SM1 (0.525 FPU/mL). Conversely, isolates SM4 (0.335 FPU/mL), SM5 (0.282 FPU/mL), and F2 (0.636 FPU/mL) displayed lower FPase activity. The distinct capacities of these isolates in cellulose breakdown are highlighted by the variety of CMCase and FPase activities; SM2, C1, and SM1 exhibit notable promise in this regard.

Protein production by fungi is of paramount importance as it relates to various cellular and extracellular functions. Fungi utilizes proteins for metabolic processes, enzyme production, and structural components, among other vital functions. Therefore, assessing protein production provides insights into the overall metabolic activity of these isolates. Isolate SM1 exhibited the highest protein production, by 0.151 mg/mL. Close behind was isolate C1, with a protein production level of 0.150 mg/mL. Other isolates, including F2 (0.127 mg/mL), SM2 (0.145 mg/mL, SM5 (0.138 mg/mL), and SM4 (0.068 mg/mL), exhibited varying degrees of protein production (Fig. 2). Fungi with higher protein production capabilities, such as SM1 and C1, are useful for enzyme production, bioremediation, and protein synthesis.

### CMCase activity and pH value of pre-treatment rice straw with fungal isolates

**Figure 3:**
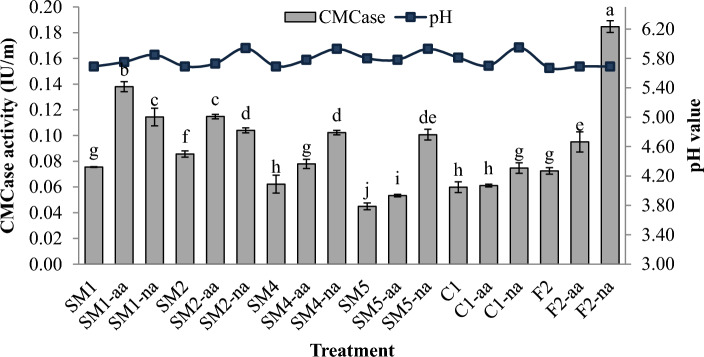
CMCase activity of fungal isolates from quantitative screening under submerged inoculation in various pre-treatment. The express as mean value ± SDTEV, the small letters indicated significant differences in values of each fungal isolate (by LSD, P ≤ 0.05).

The investigation into CMCase activity in submerged rice straw subjected to different pretreatments (5% acetic acid and 2% sodium hydroxide) in conjunction with various fungal isolates has provided intriguing insights into cellulase enzyme activity under diverse conditions (Fig. 3). Overall, both acetic acid and sodium hydroxide pretreatments demonstrated a notable enhancement in CMCase activity compared to the control condition without any treatment. This enhancement can be attributed to the selective solubilization of hemicellulose and lignin by acetic acid and sodium hydroxide, effectively disrupting their structural components^18,19^. This process results in the removal of these substrates from rice straw, thereby rendering cellulose more accessible to enzymatic degradation.

Among the fungal isolates tested, F2, SM1, and SM2 consistently exhibited outstanding enzyme release activity across various conditions. Notably, isolated F2 highlighted the highest CMCase activity, achieving significant levels following NaOH pretreatment (0.185 IU/mL). Isolate SM1 displayed robust cellulase activity, particularly following acetic acid treatment (0.138 IU/mL), surpassing its performance under both NaOH-pretreated and normal conditions. This isolate-specific variation underscores the adaptability of these fungi to different pretreatment methods and conditions. It is worth noting that the pH values of each treatment remained stable within a range of 5.67 to 5.95 throughout the study, indicating that variations in CMCase activity were primarily attributed to the pretreatment and fungal isolate combinations rather than significant pH fluctuations.

### Cellulose content (%) of rice straw inoculated with fungal isolate

**Figure 4:**
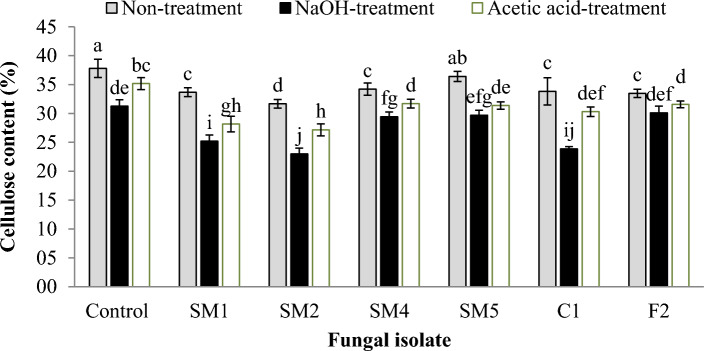
Cellulose content (%) in the rice straws substrates. The express as mean value ± SDTEV, the small letters indicated significant differences in values of each fungal isolate (by LSD, P ≤ 0.05).

The data in Fig. 4 illustrates the cellulose content (%) in rice straws and fungi substrates subjected to different pretreatment methods (NaOH and acetic acid). Pretreatment with NaOH resulted in a significant reduction in cellulose content across all fungal isolates compared to the control group. The cellulose content ranged from 23.0 to 30.1%. This decrease can be attributed to the ability of NaOH to effectively break down lignin and hemicellulose, which are components that encapsulate cellulose. Acetic acid pretreatment also led to a reduction in cellulose content when compared to the control group. The cellulose content ranged from 27.2 to 35.2%. While this pretreatment method is milder than NaOH, it still facilitates the removal of lignin and hemicellulose, making cellulose more accessible for enzymatic degradation. Fungal isolates SM1, SM2, SM4, SM5, C1, and F2 exhibited varying degrees of cellulose content reduction after both pretreatment methods. While isolates SM1, SM2 and C1 exhibited the most significant decrease in cellulose content following both NaOH and acetic acid treatments. These variations underscore the distinct capabilities of each fungal isolate in biomass degradation. The significant reduction in cellulose content after both NaOH and acetic acid pretreatment confirms the effectiveness of these methods in breaking down lignin and hemicellulose. Variability in cellulose content reduction among fungal isolates highlights their unique abilities to interact with pretreated rice straws.

### The cellulose degradation of fungi under FTIR spectroscopy

**Figure 5:**
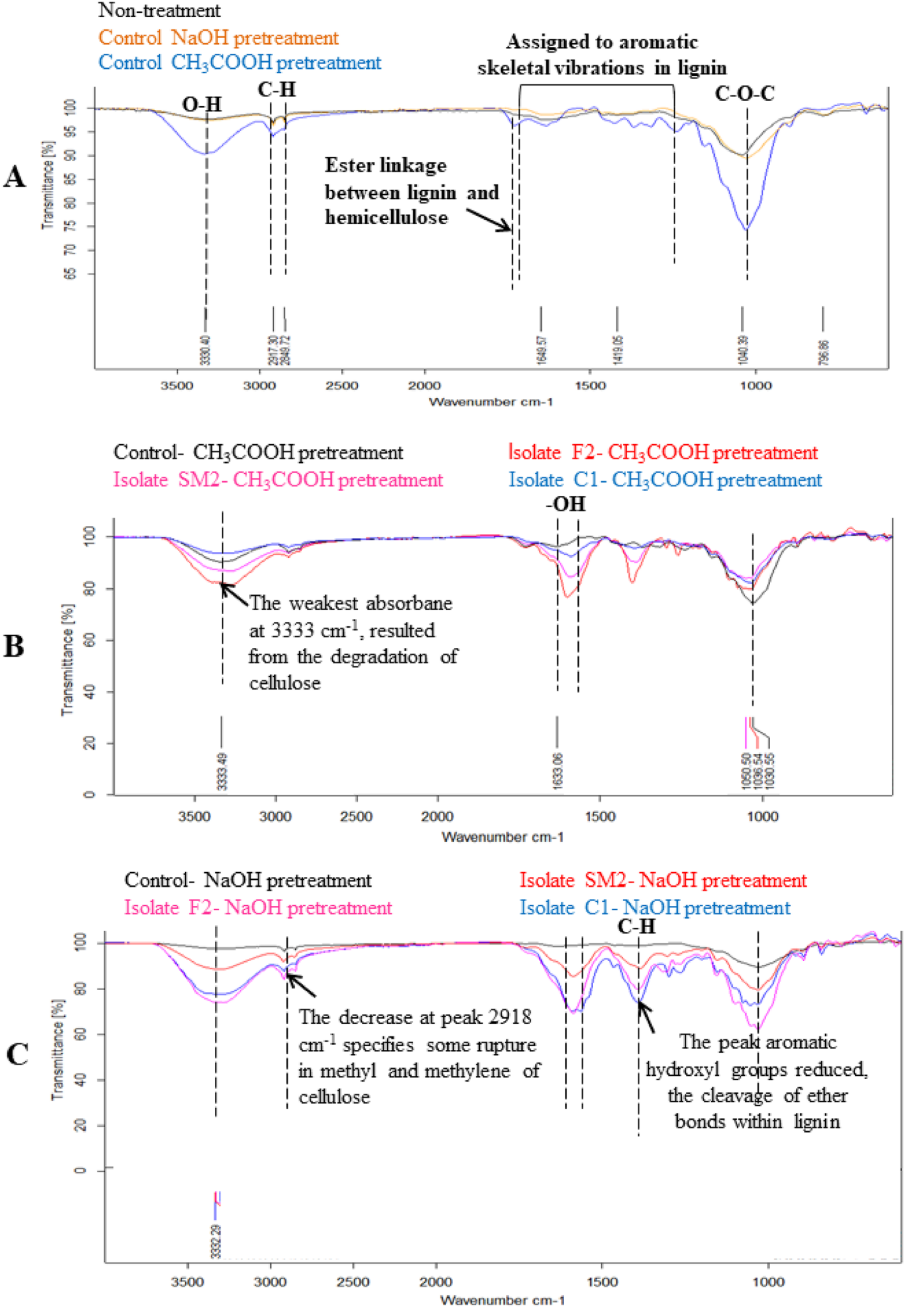
FTIR spectra of rice straw in different pretreatment method and various fungal isolate. (**A**) Control rice straw compared to NaOH or CH_3_COOH pretreatment rice straw; (**B**) CH_3_COOH pretreatment rice straw with 3 differences fungal isolates; (**C**) NaOH pretreatment rice straw with 3 differences fungal isolates.

The chemical structural changes of rice straw were determined using FTIR under different pretreatment methods (NaOH and CH_3_COOH) and fungal isolates (SM1, SM2, SM4, SM5, C1 and F2) fermentation. The FTIR spectra of samples were shown in Fig. 5, the functional group change of rice straw was particularly reflected between the absorbance spectra 400 cm^−1^ and 1800 cm^−1^. Although the FTIR spectroscopy of rice straw exhibited the same profile in all treatments (control pretreatment rice straw, pretreatment rice straw fermented with fungal isolate), the intensities of the absorption bands differed. The wide band from 3500–3100 cm^−1^ was attributed to the O–H stretching of hydrogen bonds in cellulose, hemicellulose, and lignin^20^. Figure 5A revealed the intensity decreased following NaOH and acetic acid pretreatments compared to the control raw rice straw because hemicellulose and lignin from rice straw were partially removed, which might have resulted from the degradation of cellulose. The 1649 cm^−1^ peaks presented the H–O–H stretching vibration of adsorbed water in the cellulose structure. The band at 1200 to 1100 cm^−1^ was often associated with the C–O–H stretching of cellulose and hemicellulose in rice straw, while the reduction in bands showed partial removal of lignin and hemicellulose from rice straw (Fig. 5B,C). In comparison to pretreatment rice straw, pretreatment rice straw inoculated with fungal improves cellulose removal. Furthermore, the band at roughly 1700–1720 cm^−1^ was often characterized as the C–O of ester linkages, the acetyl group in hemicellulose structure, and the coupling between lignin and hemicellulose^21^. The reduction in the band following fermentation of NaOH pretreated rice with each fungus isolate shows that these links have degraded and that new chemical groups have formed. Furthermore, the band at 1633 cm^−1^ was attributed to absorption due to C=O group deformation within the alkyl groups of the lignin side chains, and the higher reduced after rice straw treatment with NaOH and fungi compared to rice straw treated with CH_3_COOH and fungi because the alkali hydrolysis reaction may cause partial lignin structure to change from raw rice straw. The FTIR spectra results revealed that the alkaline (NaOH 2%) and acid (CH_3_COOH 5%) pretreatment rice straw addition with fungal isolate destroyed a considerable amount of lignin, cellulose, and hemicellulose. All the target fungal isolates contributed to the cellulose breakdown process in rice straw.

### Identification of fungal isolates

Based on the outstanding in cellulose degradational and plant growth promotes abilities, three selected fungal isolates were subsequently identified using ITS sequencing. The identification of the selected three isolates revealed three distinct species, namely, *Ascomycota* sp. AR-2010 isolate CY243 (HQ608067.1) (isolate SM2), *Chaetomium globosum* strain YTG2(1) (KM268672.1) (isolate C1), *Aspergillus* sp. T10 SA-2014 (KJ584849.1) (isolate F2). These three fungal isolates were further evaluated for submerged into soil amendment.

### Production of soil amendment by selected fungal and organic material

Table 3 showed the concentrations of available nutrients (nitrogen and phosphorus) and organic matter content in the soil after a 30 day incubation period with different soil additions such as rice straw, cow manure, biochar, and fungal. Treatment A6, with CaCO_3_ pre-treatment, had the greatest available N concentration on Day 7 (1.46 mg/L) and P concentration on Day 14 (1.75 mg/L). In contrast, treatment T1, the untreated control, had the lowest available N and P concentrations throughout the trial. Treatment A9, which included NaOH pre-treatment, had the greatest OM percentage on Day 21 (12.5%) compared to other treatments. Overall, treatments that included pre-treatment with CaCO_3_ or NaOH exhibited higher nutritional availability and OM content than untreated and CH_3_COOH-treated samples.

**Table 3:** Soil amendment availability of nitrogen, phosphate, and organic matter with inoculation of co-fungal isolates over 21 and 30 days.

Treatment (rice straw:cow manure:biochar:fungi)	Available N (mg/L)			Available P (mg/L)			OM (%)
	Day 7	Day 14	Day 21	Day 14	Day 21	Day 30	
A1: Untreated (5:3:2.5:0)	0.58 ± 0.01^e^	1.32 ± 0.13^b^	2.45 ± 0.13^c^	23.9 ± 0.29^a^	14.2 ± 1.33^cd^	8.29 ± 0.46^e^	0.50 ± 0.0^h^
A2: Untreated (5:3:2.5:1)	0.92 ± 0.14^cd^	0.96 ± 0.15^d^	1.91 ± 0.20^d^	7.74 ± 0.85^f^	16.7 ± 0.91^b^	12.7 ± 3.44^ab^	1.60 ± 0.07^g^
A3: Untreated (5:1.5:1:1)	0.70 ± 0.04^de^	1.29 ± 0.14^bc^	3.16 ± 0.06^a^	23.0 ± 1.15^ab^	12.9 ± 1.41^de^	11.2 ± 0.45^abc^	1.95 ± 0.0^e^
A4: CaCO_3_ treated (5:1.5:1:1)	0.74 ± 0.05^cde^	1.44 ± 0.01^b^	2.74 ± 0.15^d^	22.6 ± 0.75^ab^	14.2 ± 0.75^cd^	8.63 ± 0.21^de^	1.76 ± 0.0^f^
A5: CaCO_3_ treated (5:3:2.5:1)	0.70 ± 0.20^de^	0.99 ± 0.24^d^	2.69 ± 0.17^bc^	11.4 ± 1.65^e^	14.5 ± 0.69^cd^	13.1 ± 2.02^a^	2.16 ± 0.02^d^
A6: CH_3_COOH treated (5:3:2.5:1)	1.46 ± 0.17^a^	1.75 ± 0.02^a^	3.17 ± 0.04^ab^	18.9 ± 1.51^c^	11.4 ± 0.55^e^	10.7 ± 0.69^bcd^	2.65 ± 0.00^a^
A7: CH_3_COOH treated (5:1.5:1:1)	0.67 ± 0.02^e^	0.89 ± 0.09^d^	2.85 ± 0.06^b^	13.8 ± 1.77^d^	14.5 ± 1.17^cd^	10.0 ± 0.14^cde^	2.15 ± 0.0^d^
A8: NaOH treated (5:1.5:1:1)	0.97 ± 0.10^bc^	1.06 ± 0.22^cd^	2.91 ± 0.14^b^	22.3 ± 1.45^ab^	14.8 ± 0.20^c^	9.56 ± 0.41^cde^	2.50 ± 0.02^b^
A9: NaOH treated (5:3:2.5:1)	1.07 ± 0.27^b^	0.87 ± 0.10^d^	3.27 ± 0.16^a^	21.3 ± 0.92^b^	18.8 ± 0.94^a^	12.5 ± 0.41^ab^	2.34 ± 0.01^c^
%CV	16.33	12.41	4.98	6.76	6.52	12.87	1.44
F-test	**	**	**	**	**	**	**

To indicate significant differences at the 0.05 level, used (*); to indicate significant differences at the 0.01 level, used (**).

Small alphabetic letters indicate significant differences of each treatment by F-test (P ≤ 0.05).

### The effect of soil amendment by fungal on rice seedling

**Figure 6:**
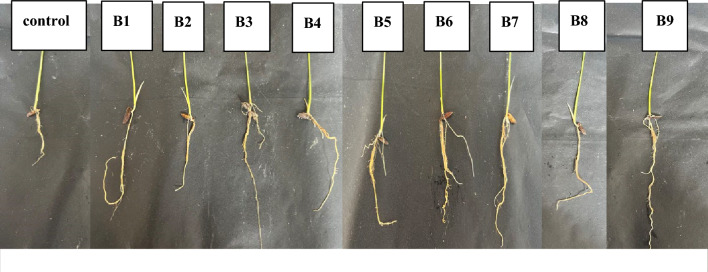
Effect of soil amendment on root elongation after 14 days in saline soil. (**B1**) Control, (**B2**) Untreated (5:3:2.5:1), (**B3**) Untreated (5:1.5:1:1), (**B4**) CaCO_3_ treated (5:1.5:1:1), (**B5**) CaCO_3_ treated (5:3:2.5:1), (**B6**) CH_3_COOH treated (5:3:2.5:1), (**B7**) CH_3_COOH treated (5:1.5:1:1), (**B8**) NaOH treated (5:1.5:1:1), (**B9**) NaOH treated (5:3:2.5:1). Ratio as follow rice straw:cow manure:biochar:fungi.

According to the experiment’s findings on rice seedling growth with soil amendment utilizing solid-state fungal fermentation, pre-treated rice straw, biochar and cow manure, treatments B6 (CaCO_3_ pre-treatment) had the greatest germination index at 70% (Table 4). This shows that adding CaCO_3_ to the soil amendment improved seed germination and early seedling development. Furthermore, B9 (NaOH pre-treatment) had the largest root length (6.66 cm) and shoot length (18.6 cm), outperformed the control treatment by 140% and 22%, respectively, demonstrating improved root system growth even under intense salt stress conditions (Fig. 6). In terms of pH and EC values, B9 significantly increased soil pH, revealing alkalization of the soil but it resulted in the lowest EC. The treated CH_3_COOH rice straw seems like inhibited the rice seedling growth, while the pH was within the range observed for other treatments. Its data gave evidence that the observed effects on plant growth are not directly attributable to changes in soil pH and salinity. Interestingly, the EC values demonstrated unexpected rice seedling development under high salt stress circumstances (EC range from 7.12 to 9.4), despite levels that normally limit yields in many crops.

**Table 4:** The effect of soil amendment by pre-treated rice straw and fungal inoculation on rice seedlings.

Treatment (rice straw:cow manure:biochar:fungi)	Germination index (%)	Root length (cm)	Shoot length (cm)	pH	EC (dS/m)
B1: Control	63.3 ± 5.7^ab^	2.77 ± 0.1^d^	15.2 ± 0.7	7.95 ± 0.0^d^	7.65 ± 0.2^ cd^
B2: Untreated (5:3:2.5:0)	36.6 ± 5.7^d^	2.88 ± 0.8^d^	15.8 ± 1.5	8.3 ± 0.0^b^	8.45 ± 0.3^abc^
B3: Untreated (5:3:2.5:1)	56.6 ± 5.7^b^	2.44 ± 0.3^d^	17.4 ± 1.5	8.32 ± 0.0^b^	9.4 ± 0.1^a^
B4: Untreated (5:1.5:1:1)	36.6 ± 5.7^d^	4.27 ± 1.1^bc^	14.2 ± 1.8	8.2 ± 0.0^bc^	8.39 ± 0.1^abc^
B5: CaCO_3_ treated (5:1.5:1:1)	53.3 ± 5.7^bc^	4.22 ± 0.5^bc^	16.5 ± 1.5	8.23 ± 0.0^bc^	8.9 ± 1.2^ab^
B6: CaCO_3_ treated (5:3:2.5:1)	70 ± 10^a^	4.55 ± 0.7^b^	17.8 ± 1.8	8.17 ± 0.0^bc^	9.3 ± 0.2^a^
B7: CH_3_COOH treated (5:3:2.5:1)	43.3 ± 5.7^ cd^	3.33 ± 0.5^ cd^	16.7 ± 1.9	8.01 ± 0.1^d^	8.13 ± 0.0^bcd^
B8: CH_3_COOH treated (5:1.5:1:1)	43.3 ± 5.7^ cd^	4.55 ± 0.3^b^	14.1 ± 1.0	8.2 ± 0.0^bc^	9.15 ± 0.2^ab^
B9: NaOH treated (5:1.5:1:1)	70 ± 10^a^	6.66 ± 0^a^	18.6 ± 0.3	8.48 ± 0.0^a^	7.12 ± 0.6^d^
B10: NaOH treated (5:3:2.5:1)	60 ± 10^ab^	4.33 ± 0.8^bc^	16 ± 0.5	8.08 ± 0.0^ cd^	9.1 ± 0.2^ab^
%CV	13.69	16.1	10.64	0.78	5.79
F-test	**	**	ns	**	*

To indicate significant differences at the 0.05 level, used (*); to indicate significant differences at the 0.01 level, used (**).

Small alphabetic letters indicate significant differences of each treatment by F-test (P ≤ 0.05).

## Discussion

Microbial communities, which include fungi and bacteria, play a significant role in soil ecosystems by regulating nutrient cycling, organic matter breakdown, and soil structure creation, all of which are essential for maintaining soil fertility and sustaining plant growth^19^. The isolation and characterization of microorganisms from varied environments provides prospects for their use in a variety of biotechnological applications. Microorganisms from saline or semi-saline soils, for example, may include salt-tolerant mechanisms or enzymatic activities that might help remediate saline soils or improve crop salinity tolerance. Similarly, microorganisms isolated from carbon and filter cake powders may break down organic pollutants or facilitate composting processes in agricultural waste management^22^. Notably, fungal isolates such as *Chaetomium globosum* and *Aspergillus* sp., derived from carbon and filter cake, have exhibited extraordinary efficacy in rice straw degradation, as proven by our study’s excellent results.

Understanding fungal isolates’ pH preferences and performance has important implications for environmental management and biotechnology, particularly in terms of maximizing their use in environmental remediation, agriculture, and industrial processes. Our investigation found that the fungal isolates investigated in this study had a wide pH tolerance, spanning from acidic to alkaline. For example, fungal isolates *Chaetomium globosum* C1 and *Aspergillus* sp. F2 thrived in alkaline conditions, instead *Ascomycota* sp. SM2 grew consistently over a pH range of 4 to 9. These results highlight the significance of pH in microbial ecology and bioprocess optimization, highlighting the necessity of taking pH dynamics into account while using their potential, which supported by study of Mustafa et al.^22^.

In our evaluation of rice growth, we propose that our soil amendment, which includes fungal isolates, cow manure, and biochar, might mitigate the harmful effects of salt stress in the environment. Kumari et al.^23^ suggested that the use of organic amendments, fungus, biochar, and vermicompost can successfully reduce the negative effects of salinity on plants. Furthermore, Evelin et al.^24^ found that *mycorrhiza*l fungus can help plants cope with salt stress. *Chaetomium globosum* has been found as a salt-tolerant fungus while *Aspergillus* possesses both salt-tolerant and thermostable qualities, making it beneficial for industrial applications and lignocellulosic consumption^25,26^. Likewise, the use of biochar increases salt buildup at the soil surface, resulting in a more favorable salinity level for plant development below ground while also aiding mechanical desalination^27,28^. Consequently, our study detected an expansion in root development, even under acute salt stress circumstances.

Cellulases are essential for organic matter decomposition and nitrogen cycling in soil conditions^29^. Our investigation highlighted the significant role of cellulase-producing fungal isolates, including fungal isolates *Aspergillus* F2, *Ascomycota* sp. SM2, and *Chaetomium globosum* C1, in efficient cellulose breakdown, as evidenced by CMCase and FPase activity. These isolates show potential for several uses, such as enzyme synthesis and bioremediation, due to their strong protein production capabilities^28,30–33^. *Chaetomium* sp. is commonly found in interior areas and can degrade cellulose-based construction materials, potentially causing structural damage^28^. Despite the limited knowledge offered, this study has demonstrated the potential of *Ascomycota* sp. in the breakdown of cellulose, a finding corroborated by research published by Pertile et al.^34^. Furthermore, fungal isolates revealed the ability to enhance plant development through nutrient solubilization, and IAA synthesis, highlighting their multifunctionality in soil^35–38^.

The improvements in CMCase illustrates fungal after acetic acid and sodium hydroxide pretreatment of rice straw illustrate the effectiveness of these methods in improving cellulose accessibility for enzymatic breakdown. These pretreatments preferentially dissolve hemicellulose and lignin of rice straw, increasing cellulose accessibility and allowing for more effective cellulase enzyme activity^33^. NaOH’s strong alkaline properties effectively destroy lignin and hemicellulose structures, resulting in a considerable fall in cellulose content. Boodaeng et al.^39^ found that pretreatment of rice straw with diluted alkali resulted in higher reducing sugar yields than acid treatment. FTIR spectroscopy revealed considerable structural changes in rice straw throughout pretreatment and SSF by fungi, showing the degradation of lignin, cellulose, and hemicellulose. These findings highlight the potential of fungal isolates such as *Ascomycota* sp. SM2, *Chaetomium globosum* C1, and *Aspergillus* sp. F2 for biomass conversion processes, providing important insights into the efficacy of acid and alkaline pretreatment techniques in biotechnological applications. Despite the technological limitations provided by the low bioavailability of lignocellulosic residues^40^, selecting sufficient pretreatment procedures for these resistant substrates is critical for improving biodegradation efficiency. FTIR research demonstrated that white-rot fungi promote lignin breakdown in cell walls by oxidizing aromatic rings, which supports recent chemical investigations by Bari et al.^41^.

SSF by fungi provides an excellent manufacturing technique for cellulase, delivering reduced costs and greater enzyme titers^42^. In this study, the addition of fungus to soil amendments tended to improve nutrient availability, especially in treatments with NaOH and CaCO_3_-treated rice straw. Fungal-mediated decomposition mechanisms are thought to have accelerated the breakdown of organic materials, resulting in prolonged nutrient release throughout the incubation period. The observed differences in nutrient availability and organic matter content highlight the significance of soil amendment ingredient choices in agricultural activities. Microbial activity is often strong during the early stages of decomposition (Day 7), allowing for the fast breakdown of easily accessible organic molecules. As a result, there is an initial surge in nutrient release as microbial biomass grows and organic matter breakdown accelerates. However, as decomposition advances, the supply of easily decomposable organic matter decreases, reducing microbial activity and corresponding nutrient release rates. This drop is visible in the middle to late stages of the trial (Day 14 onwards). Furthermore, when microbial populations settle and attain a stable state, nutrient mineralization rates may slow, resulting in a reduction in nutrient release into the soil solution. The present investigation reveals that soil amendment using SSF of NaOH or CaCO_3_-pretreated rice straw in combination with fungal inoculation can improve soil fertility and plant development. However, while this strategy enhanced rice development, the resulting pH values may not be ideal for certain plant species that need slightly acidic to neutral soil conditions. The addition of soil additives raised the soil pH since the research began with alkaline soil. Fungi release enzymes and create organic acids including oxalic acid, citric acid, and acetic acid as organic matter decomposes^43^. They also emit alkaline compounds like ammonia and different basic minerals as metabolic byproducts. The total effect of fungal breakdown on soil pH is determined by the balance between the generation of these chemicals and the alkalinity introduced by the NaOH pretreatment procedure. As a result, it is claimed that our soil amendment, which includes NaOH and CaCO_3_-pretreated rice straw and fungal inoculation, may assist in reducing soil acidity by gradually moving the pH to a more neutral or slightly alkaline level. This change might have a favorable impact on plant development and overall soil health in agricultural contexts.

## Conclusion

In conclusion, the fungal strains *Ascomycota* sp. SM2, *Chaetomium globosum* C1, and *Aspergillus* sp. F2 showed considerable cellulase activity, aiding rice straw decomposition while also stimulating rice plant development. This work sheds light on how fungal isolates, pretreatment techniques, and SSF processes improve soil fertility, promote plant growth, and facilitate biomass breakdown. These discoveries advance our understanding of microbial ecology, biotechnology applications, and the adoption of sustainable farming methods. However, further study is required to determine the impact of various fungal additions on soil remediation, as well as the mechanistic interactions between fungal enzymes, rice straw components and soil dynamics.

## Materials and methods

### Sample collection and microorganisms screening

Saline soil, rice straw was collected from rice field in Daeng Yai, Mueang Khon Kaen district, Khon Kaen province (Thailand). Filter cake powder and biochar were purchased from a market in Khon Kaen province. The geographical coordinates are at 16.4837° N latitude and 102.7480° E longitude. All methods were performed in accordance with relevant guidelines and regulations.

Fungal isolation was done by serial dilution method. One gram sample (heavy saline soil, semi-saline soil, filter cake powder, and biochar powder) was suspended in 10 mL sterile distilled water. An aliquot of diluted sample was spread plated onto a potato dextrose agar (PDA), and lactic acid (85%) was added to the medium (l g/L) to stimulate the growth of fungi, then the agar plates were at 28 ℃ until fungal growth was evident. The differences in morphology microorganisms were collected and purified. The microorganisms were maintained on PDA at 4 ℃ for further study.

### Plant growth promoting assay

All isolates were cultured in nutrient broth (NB) medium supplemented with l-tryptophan (500 mg/L) for IAA production test^44^. Salkowski’s reagent (2% 0.5 M FeCl_3_ in 35% HClO_4_) was added to the supernatant and read at 536 nm under spectrophotometer. For potassium and phosphates solubilization test, isolates were spotted on Aleksandrov agar medium and National Botanical Research Institute Phosphate (NBRIP) agar medium, respectively^45^, incubated for 5 to 7 days at 28 ℃, then measured the solubility index.

### Enzymatic screening

All fungal isolates were screened for visual cellulolytic activity using 2% (w/v) carboxy methyl cellulose (CMC) agar medium. A fungal plug was inoculated aseptically in the center of CMC agar plates and incubated at 28 ℃ for 5–7 days. The plates were examined to produce clear halo zones on CMC plate when it was treated with 1% Congo red dye for 15 min and rinsed with 1 M NaCl.

### The effect of pH on fungi growth

Selected fungal isolates were tested for their growth capability across various pH levels. The different pH levels of nutrient agar (NA) medium were prepared and adjusted using hydrochloric acid (0.1 M HCl) or sodium hydroxide (0.1 M NaOH). The pH level was measured using an electrical pH meter before sterilization in an autoclave at 121 ℃. Fungal isolate was measured fungal diameter and biomass after 15 days of incubation.

### Enzyme assay

#### Endoglucanase assay

The fungal extract was prepared from fungal cultured in a NB medium. After filtering, the fungal biomass was ground by liquid nitrogen. A buffer solution (50 mM sodium citrate (pH 4.8) and 0.02% Triton X-100) was added to grind biomass. The crude enzyme was collected by centrifugation at 8000 rpm at 4 ℃.

Endoglucanase activity was measured according to the procedure described by Ghose et al.^46^. The 3, 5-Dinitro Salicylic Acid (DNS) chemical was used for determining reducing sugars. The reaction of the mixture contained 0.5 mL of 1% CMC in 0.05 M Na-acetate buffer (pH 5) and 0.5 mL of appropriately diluted crude enzyme. The mixture was incubated at 50 ℃ for 1 h. The reducing sugar released was estimated by the addition of 3, 5-dinitrosalicylic acid (DNS) with glucose as standard. The absorbance was read at 540 nm using a spectrophotometer.

$$CMCase\frac{IU}{\text{mL}}=\frac{0.185}{enzyme\, concentration \,required \,to \,release\, 0.5\,{\text{mg}}\, of \,glucose}.$$

#### Cellulase (FPase) assay

The cellulase activity was determined using the filter paper asset with a Whatman No.1 filter paper strip equivalent to 50 mg of substrate according to Ghosh et al.^46^. The suitable dilution was made up. The reaction mixture contained 1 mL of 0.05 M Na-citrate (pH 5), the filter paper trip, and 0.5 mL of crude enzyme diluted accordingly. The mixture was incubated at 50 ℃ for 1 h, and a 3 mL DNS was added and read at 540 nm by spectrophotometer. The released reducing sugar was estimated based on glucose standards.

$$FPase\frac{IU}{\text{mL}}=\frac{0.37}{enzyme\, concentration\, required \,to\, release\, 2 \,{\text{mg}}\, of\, glucose}.$$

#### Protein concentration assay

The extracellular protein concentration was determined by the Bradford method^47^. A 50 µL fungal extract was added by 950 µL Bradford reagent (1 litre: 100 mg Coomassie Brilliant Blue G250 in 50 mL 95% ethanol, 100 mL 85% phosphoric acid, filtered through 0.2 µm membrane filter). After 10 min incubation at room temperature, the sample was read at 595 nm by spectrophotometer and compared to the standard curve of bovine serum albumin (BSA).

### In vitro cellulase enzyme production under solid-state fermentation (SSF) conditions

Fungal isolates capable of plant growth promotion and cellulose degradation were chosen for enzyme production under SSF conditions. Rice straw was pretreated by two different methods, one with NaOH 2% for 1 h, and another with acetic acid 5% for 1 h. Rice straw was washed until neutral pH and air dried for 2 days. After that, 5 g of rice straw was added into the flask with 50 mL of nutrient medium (2 g (NH_4_)_2_SO_4_, 2 g KH_2_PO_4_, 0.3 g MgSO_4_, 0.3 g CaCl_2_, 0.5 g NaCl, 0.005 g FeSO_4_, 0.016 g MnSO_4_, 0.017 g ZnCl_2_). These were autoclaved at 121 ℃ for 15 min. The fungal isolates were grown on NA medium for 5–7 days before harvesting the spores in sterile saline. The obtained spore solution was dispersed in saline, and 1 mL of it was diluted with 9 mL of saline before measuring the optical density (OD) at 600 nm with a spectrophotometer. A hemocytometer was used to determine and modify the spore concentration of the fungal isolates based on their OD value of 1^48^. Subsequently, fungal suspension was added to the flasks and incubated at 28 ℃ for 30 days. For extraction of enzyme activity, 50 mL of citrate buffer (50 mM, pH 4.8) was added and shaken for 30 min. The contents were filtered, and the filtrate was centrifuged at 8000 rpm for 20 min. The final filtrate was collected and measured the enzyme activity following the enzyme assay as mentioned above.

### Cellulose content (%)

The cellulose content from rice straw was preformed according to a method described in the study of Luo et al.^49^ with some modified. One gram of dry biomass was fluxed for 20 min with 10 mL of 80% acetic acid and 1.5 mL of nitric acid. The mixture was dried in hot air oven at 105 ℃ until it reached a constant weight, and the difference between the initial and final weights was used to calculate the content of cellulose (%)

$$Cellulose\, content \,\left(\%\right)=\frac{final\, weight-initial\, weight}{total \,weight}\times 100.$$

### Cellulose degradation identification through infrared spectroscopy by Fourier transformer (FTIR)

The FTIR spectrum was carried out in a spectrometer FTIR (brand) which has a resolution of 4 cm^−1^ to characterize the functional groups of the samples, the sample was finely ground and pre-dried KBr before reading by FTIR. The region used for the analysis was 400–4000 cm^−1^.

### Identification of fungal isolates

Fungal strains were incubated on the NA medium at 28 ℃ for 5–7 days, the colonies were characterized by the morphological method. The total genomic DNA of fungal strains were extracted by the CTAB method^50^. The molecular identification of fungal strains was carried out based on the analysis of internal transcribed spacer (ITS) region sequences using universal primers ITS1. Following sequencing, the Basic Local Alignment Search Tool (BLAST) software was utilized to align the gene sequences of various bacterial strains with bacterial sequences within the National Center for Biotechnology Information (NCBI) databases.

### SSF-assisted production of soil amendment using selected fungal isolates and organic materials

#### Formula preparation

Rice straw was initially pre-treated by soaking in calcium carbonate (CaCO_3_), acetic acid (CH_3_COOH), or sodium hydroxide (NaOH) for 20 min to enhance the degradation process. Co-inoculated 3 fungal isolates with 20 ml of spore suspension (fungal spore prepared as follow method of Singh et al.^48^) were supplemented to soil amendment. Then, soil amendment was prepared by two formulas as follows the Table 5. The substrates were contained in the cup with a well cover and incubated at 28 ℃ for 30 days.

**Table 5:** The formula of soil amendment of rice straw degradation by fungi.

Pre-treatment	Material	Formula 1	Formula 2
Un-treated/CaCO_3_ treated/CH_3_COOH treated/NaOH treated	Rice straw (g)	100	100
	Cow manure (g)	32	62
	Biochar (g)	25	50
	Fungal isolate (ml)	20	20
	Water (g)	50	50
	Total (g)	227	282

#### Characterization of soil amendment substrate by rice straw and *fungi*

Using a pH meter and an EC meter to measure the pH and EC values of each rice straw amendment formulation were evaluated in water extracts (follow the ratio of 1:1 for pH and 1:5 for EC).

The available of nitrogen and phosphorus content were assessed using the Kjeldahl method. The colorimetric method with molybdenum was employed to extract and measure the available phosphate (orthophosphate) in rice straw substrates^51^.

#### Seedling experiment design and measurement of plant growth parameters

Rice seedlings were provided by Salt-tolerant Rice Research Group, located within Faculty of Science, Khon Kaen University, Thailand (16° 28′ 15.82′′ N, 102° 49′ 11.48′′ E). The study used a completely randomized design with five replications. Seven-day-old rice seedling was transferred to a pot containing 100 g soil and 5 g substrates. All methods were performed in accordance with relevant guidelines and regulations. Rice seedlings have grown under greenhouse conditions for 14 days with full irritation. Rice plant height and germination index of 14 day-old rice were determined. Organic matter analysis was performed using the method outlined by Walkley et al.^52^.

### Statistical analysis

Statistic 10 software was used to run the ANOVA analysis on the data. The Least Significant Difference (LSD) technique was also used to differentiate mean values at 95% and 99% significant.

## Data Availability

All data in this study are available upon request from the corresponding author at nunrid@kku.ac.th.
